# Assessment of Knowledge, Attitude and Practice towards Prevention of Respiratory Tract Infections among Hajj and Umrah Pilgrims from Malaysia in 2018

**DOI:** 10.3390/ijerph16224569

**Published:** 2019-11-18

**Authors:** Mohammed Dauda Goni, Habsah Hasan, Nyi Nyi Naing, Nadiah Wan-Arfah, Zakuan Zeiny Deris, Wan Nor Arifin, Aisha Abubakar Baaba

**Affiliations:** 1 Department of Medical Microbiology and Parasitology, School of Medical Sciences, Universiti Sains Malaysia Health campus, Kubang Kerian 16150, Malaysia; dmgoni@yahoo.com (M.D.G.); zakuan@usm.my (Z.Z.D.); 2 Faculty of Medicine, Medical Campus Universiti Sultan Zainal Abidin, Kuala Terengganu 20400, Malaysia; 3 Faculty of Health Sciences, Gong Badak campus, Universiti Sultan Zainal Abidin, Kuala Nerus 21300, Malaysia; wanwaj@unisza.edu.my; 4 Unit of Biostatistics and Research Methodology, School of Medical Sciences, Universiti Sains Malaysia Health Campus, Kubang Kerian 16150, Malaysia; wnarifin@usm.my; 5 Centre for Language Studies and Generic Development, Universiti Malaysia Kelantan, Locked Bag 01, Bachok 16300, Kelantan; mandybaaba@gmail.com

**Keywords:** knowledge, attitude, practice, respiratory tract infections, pilgrims, Malaysia

## Abstract

Respiratory tract infection (RTI) is a major public health challenge during the Muslim pilgrimage to Makkah. This study aims to evaluate the knowledge, attitude, and practice of Malaysian Hajj and Umrah pilgrims towards the prevention of RTIs in 2018 and determine correlations among three domains. A cross-sectional study was conducted among 225 Umrah and Hajj pilgrims. Knowledge, attitude, and practice (KAP) towards RTI prevention was assessed by using a validated self-administered questionnaire among pilgrims attending a weekly orientation course organized by private Hajj/Umrah companies. Out of 225 participants, 65.9% of respondents were female with the mean (SD) age of 46.74 (13.38) years. The interquartile range (IQR) score for knowledge is 18.0 (6.0), the mean scores of attitude and practice are 32.65 (4.72) and 25.30 (4.9). respectively. Significant and negative linear correlations between knowledge and practice (*r* = −0.232, *p* < 0.001), and attitude and practice (*r* = 0.134, *p* = 0.045) were observed. Results from the current study showed good knowledge of RTIs among Malaysian pilgrims. However, a poor attitude was reflected in their preventive practice behaviors. This will further help in the prevention and management of RTIs during Hajj and Umrah. Therefore, an extensive educational health campaign should be provided to pilgrims to create awareness.

## 1. Introduction

The Muslim pilgrimage to Mecca in Saudi Arabia is among the five pillars of the religion of Islam and is obligatory to each financially and able-bodied Muslim to perform it at least once in a lifetime. Umrah, also known as Lesser Hajj, can be performed at any time of the year and is not obligatory. This pilgrimage attracts millions of worshippers for Umrah and about two to three million people from various countries across the globe converge for the yearly Hajj rituals [1]. The official Hajj quota for Malaysian pilgrims stands at 30,200 based on the report from the Malaysian Hajj Fund [2].

Respiratory tract infections are the most common infections reported among pilgrims during Hajj and Umrah [3]. The majority of pilgrims develop one form of respiratory tract infection or another during their stay in Makkah and Madinah [4]. Various adverse conditions like overcrowding, extreme climatic condition, pollution, shared accommodation characterize these pilgrimages. Similarly, most of the pilgrims are older people (>50 years) with comorbidities such as hypertension, diabetes, and liver diseases [5,6]. These scenarios could increase the risk of spread and transmission of infections, particularly respiratory tract infections (RTIs) [7]. Respiratory pathogens and symptoms are high among Malaysian Hajj pilgrims and range from 58.9% to 94.3% [8,9,10].

Pilgrims’ knowledge, attitude, and practice are regarded to have a significant influence on the understanding of uptake of preventive measures and bridging the gap towards delivering of health information [11,12]. The gap in knowledge, as well as the poor attitudes and bad practices in relation to disease prevention and control, contribute to the increased risk of infection and negative impacts during mass gatherings [13]. The assessment of pilgrims KAP towards respiratory tract infections and their preparedness have received considerable attention from many researchers from different countries across the globe [14,15,16,17,18]. To ensure effective preventive practice and optimum preparedness regarding respiratory tract infection prevention during Hajj and Umrah, data on pilgrims’ knowledge, attitude, and preventive practices (KAP) are required. This information would be significant in providing the needed strategies to be implemented. Moreover, these would enhance and evaluate the presently available programs as well as recognize possible interventions to improve the behavioral and attitudinal changes. Positive attitude and behavioral changes are driven by the level of knowledge and perceptions towards preventive practices [19]. However, there is no study conducted in Malaysia to assess the KAP of both Umrah and Hajj pilgrims. Therefore, this study is aimed to evaluate knowledge, attitude and practice regarding the prevention of respiratory tract infection and to explore the relationship between the demographic variables and knowledge, attitude and practice scores among 2018 Malaysian Hajj and Umrah pilgrims.

## 2. Materials and Methods

### 2.1. Study Design

A cross-sectional study was conducted among Umrah and Hajj pilgrims attending weekly Hajj and Umrah orientation course organized by private Hajj/Umrah companies between July and November 2018. Participating in the research was voluntary and anonymous. Hajj/Umrah pilgrims from Malaysia, 18 years old and above, with the ability to read and write were considered eligible for the study. The sample size was determined using a margin of error of 5%, a confidence interval (CI) of 95%, and an expected response rate of 70% to most of the main questions. The minimum sample size estimated for the study was 188. We enrolled a larger sample size of 225 pilgrims in accounting for errors and non-respondents.

### 2.2. Sampling Method

The sampling method used was cluster sampling, which was done in two stages. The first stage was a purposive selection of Hajj and Umrah companies as clusters. The second stage was by applying a convenience sampling method. The pilgrims who attended the orientation course were selected as the study participants. An honorarium was given to the respondents for participating in the study. All participants involved in the study were briefed about the study at the beginning of orientation. Verbal consent was obtained from the participants, followed by the administration of the KAP questionnaire. Only those who fulfilled the inclusion and exclusion criteria and consented were included in this study.

### 2.3. Study Population

Hajj/Umrah travel companies were eligible if the management was willing to participate actively in the study and to collaborate with the researchers from Universiti Sains Malaysia. Nine eligible Hajj/Umrah companies were identified in Kota Bharu, Kelantan. All nine companies were contacted and informed about the project, and they agreed to participate. From the two companies, the majority of the participants are Umrah pilgrims 89.7% (202/225) and only 10.3% (23/225) are Hajj pilgrims.

### 2.4. Measurement Tool

A self-administered questionnaire was developed and validated by a panel of experts consisting of an epidemiologist, microbiologist, health educationist, and medical statistician to collect valuable information and KAP regarding prevention of respiratory tract infection and included questions about vaccination history and previous Hajj and Umrah experience. The questionnaire was initially drafted in English language and then underwent the “forward-backward-forward” translation from English into Malay and then to English again by another translator all from the School of Languages Literacies and Translation, Health Campus Universiti Sains Malaysia. A bilingual expert did the translation aimed to maintain the content meaning over a word for word literal translation. Back translation to the original language version (English) was done without access to the initial English version questionnaires. The questionnaire was then assessed for equivalency to the original version and the back-translated version and ascertained it to be satisfactory by a panel of experts. No significant modifications were identified, showing that the scale was adequate to maintain its meaning and purposes. It also collected participants’ demographics including age, gender, occupation, marital status, educational qualifications, as well as comorbidities and signs and symptoms of influenza-like illness prior to departure to Hajj or Umrah. The difficulty discrimination index of the knowledge domain comprising of six sub-domains ranged from −1.26 to 6.29, while the factor loading of the attitude domain which consisted of two sub-domains ranged from 0.414 to 0.791. The practice domain also consisted of two sub-domains and had factor loading range from 0.315 to 0.917. The questionnaire was piloted among 50 pilgrims before Hajj and validated. Results of the reliability test carried out showed Cronbach’s coefficient alpha for knowledge, attitude, and practice was 0.777, 0.709, and 0.729 respectively. The newly developed questionnaire had 79 items divided into five sections: Socio-demographic, knowledge, attitude, and practice sections. The knowledge section consisted of 29 items and was aimed at accessing and evaluating the general knowledge of pilgrims about the etiology, spread, and transmission, signs and symptoms, complications, risk factors and prevention of respiratory tract infections. In the attitude section, 13 questions were used to assess behavioral perception towards prevention of respiratory tract infections. Twelve questions on practices were used to evaluate the actual compliance and uptake of various preventive measures.

For the knowledge questions, incorrect or uncertain (do not know) responses were given a 0 score, while 1 point was given for choosing the correct answer. The expected maximum total knowledge score was 29. For the attitude and practice sections, a score of 1 was given for choosing the answer reflecting a positive attitude or good practice and 0 was given for choosing the answer reflecting negative attitude or poor practice. The expected maximum total attitude score is 65 and a minimum score of 13. A correct statement with options strongly agree, agree, not sure, disagree, and strongly disagree are scored 5, 4, 3, 2, and 1, respectively. Practice are scored 2, 1, and 0 for “always”, “occasional” and “never” respectively. Pilgrims’ KAP levels were defined as “good” or “poor” based on Bloom’s cut off point. Pilgrims with knowledge scores above 60% were regarded as having good knowledge, while those who scored below 60% were considered having poor knowledge [20]. Pilgrims with attitude scores of 80% and above were grouped as those with a good attitude, while pilgrims within the range of 60–79% are considered moderate and score below 59% are regarded as having an unacceptable attitude [21]. For practice section, participants with scores >80% were classified as having acceptable preventive practice, while those with scores <80% were considered having unacceptable preventive practice [22].

### 2.5. Ethics Approval and Consent to Participate

Ethical approval was obtained from the Human Research Ethics Committee of Universiti Sains Malaysia (ref no: USM/JEPeM/17020146) and Universiti Sultan Zainal Abidin (UniSZA) Human Research Ethics Committee (UHREC) (UniSZA/UHREC/2019/88) before this study. The questionnaire was designed to be anonymous, and informed consent was obtained from every respondent. The data were kept confidential and the results did not identify the respondents personally. 

### 2.6. Data Analysis

Data entry and statistical analysis were performed using the Statistical Package for Social Sciences (SPSS) version 24.0 (SPSS, Inc., Chicago, IL, USA) and were presented by using mean and standard deviation. Categorical variables were presented by using frequency and percentage. Descriptive statistics were used to determine the socio-demographic factors and KAP scores. The KAP assessment was carried out by assigning scores to the variables. To assess the role of socio-demographic characteristics on KAP, differences in socio-demographic status were compared with the KAP scores using ANOVA or independent sample t-test as appropriate. Pearson correlation coefficient was used to describe the strength and direction of the relationship among knowledge, attitudes, and practices (KAP). Correlations were interpreted using the following criteria: 0–0.25 = weak correlation, 0.25–0.5 = fair correlation, 0.5–0.75 = good correlation and greater than 0.75 = excellent correlation [23].

## 3. Results

### 3.1. Socio-Demographic Data

The ages of the respondents ranged from 18 to 74 years, with a mean (SD) age of 46.74 (13.38) years. The majority of the respondents were of Malay ethnicity 223 (99.1) and there were 151 (67.1) females. Most of the respondents 169 (75.1%) were married at the time of the research, while single and divorced/widowed accounted for 46 (20.4%) and 10 (4.4%), respectively. The education level of the respondents was secondary school level 80 (35.6%) followed by bachelor’s degree holders 78 (34.7%). Occupation status of the respondents revealed that civil servant accounted for 95 (42.2%), pensioners 41 (18.2%), and 31 (13.8%) housewives. The summary of the characteristics is shown in Table 1.

### 3.2. Assessment of Pilgrims’ Knowledge towards Prevention of RTIs

Out of the 225 participants, 126 (56.0%) and 99 (44.0%) of the participants were within the satisfactory and unsatisfactory knowledge range, respectively. The median and interquartile range (IQR) of the total knowledge score for the baseline study participant was 18.0 (6.0). Most of the respondents were aware of the etiology, risk factors, and transmission of respiratory tract infections, as summarized in Table 2. However, 54.2% of the pilgrims held misunderstanding that allergies caused RTIs. Only 23.1% and 25.8% of the participants were aware that RTI could result in multi-organ failure and breathing difficulties, respectively. Knowledge was good for responses pertaining to questions relating to the effectiveness and re-use of a face mask. The participants also knew that respiratory tract infections could be transmitted through the air (85.3%). Surprisingly, 60.9% of the participants thought RTIs could be spread by water. The correct answers to questions on the effectiveness of a cloth face mask compared to a two-ply surgical face mask and the re-use of used face masks stored in bags for later use were 77.3 and 76.4%, respectively. Regarding the knowledge of preventive practices of RTIs, most of the participants (81.3%) knew RTIs could be prevented by following a healthy diet (89.8%), receiving vaccinations (83.1%), washing hands with hand sanitizers (87.1%) and wearing a face mask (90.7%). However, covering the nose with hands as a protective measure was known by only 32.0% of the participants.

### 3.3. Pilgrims’ Attitude Towards Prevention of RTIs

The majority of the pilgrims had an unacceptable attitude (93.8%) towards the prevention of respiratory tract infections, while few (6.2%) of the participants had a moderate acceptable attitude based on the mean total attitude score. The mean (SD) of the attitude scores was 32.65 (4.72). Forty-five (20.0%) of the participants strongly agreed that since the bird flu, SARS, MERS-COV, and H1N1 crises were over, they no longer needed to worry about contracting flu-like illnesses, as shown in Table 3. However, regarding attitudes towards face mask, only 5.8% and 1.3% of participants disagreed and strongly disagreed with the opinion of being opposed to face mask usage, respectively.

### 3.4. Pilgrims’ RTIs Prevention Practice

Table 4 shows the responses of the participants regarding the various preventive practices using 13 questions. The mean (SD) of the total practice score of the participants is 25.30 (4.9). Among the pilgrims, 38 (16.9%) had a good practice and 187 (83.1%) had a poor practice towards respiratory tract infection prevention. The majority of the pilgrims occasionally eat vegetable and fruits (61.8% and 63.6%, respectively). However, more than half of them practiced protective measures in their hygiene, including handwashing after touching personal items of people with cough, shaking people’s hands and touching doorknobs, and after coughing or sneezing. About 24.0% of the respondents always refrain from being close to those that cough or sneeze. However, less than half of the respondents reported practicing receiving a flu vaccine.

### 3.5. Association of Demographic Characteristics and Mean KAP Scores

The relationship between demographic characteristics and mean KAP scores of the participants are shown in Table S1. Among the demographic variables, the gender of the respondents was significantly associated with both mean attitude and practice scores. All the demographic variables under study were significantly associated with mean KAP scores (*p* < 0.05) except for ethnicity and marital status.

### 3.6. Correlation between KAP Scores

The correlation revealed significant negative linear correlations between knowledge and attitude (*r* = −0.059, *p* = 0.378), as shown in Figure S1. Figure S2 shows the correlation between knowledge and practice (*r* = 0.232, *p* < 0.001), and attitude and practice (*r* = −0.134, *p* = 0.045), as shown in Figure S3. The result reaffirms a weak correlation between knowledge, attitude, and practice with respiratory tract infections KAP scores.

## 4. Discussion

This current study, which is believed to be the first of its kind among Malaysian Hajj and Umrah pilgrims sought to assess their KAP towards prevention of RTI. Several studies have been documented to show the KAP of pilgrims from many countries towards the prevention of RTIs [15,24,25]. However, data on the knowledge, attitudes, and preventive practices toward respiratory tract infections among Malaysian Hajj pilgrims are still lacking. With an increasing prevalence of respiratory tract infections among pilgrims during Hajj and Umrah, there is a critical need to gather essential data for effective control and preventive plans.

Results of this study revealed a good KAP score (56.0%) among the study participants towards RTIs. The median knowledge score was 18.0 (6.0) which also indicates a good level of knowledge. These results corroborate the findings of a study conducted among American Hajj pilgrims [26,27]. Only 20.0% of the participant believed that viruses do not cause RTI. A good percentage of respondents had good knowledge about the transmission of RTI. These results are in line with the findings from Tashani et al. [18], where 58% of the pilgrims reported correct means of transmission. Contrary to the findings in this study about knowledge of RTIs, several studies reported a low level of knowledge about respiratory tract infections [15,16]. The responses based on the transmission of RTIs through water is 39.1%, by sharing towels with an infected person is 51.6% and through the air is 85.3%. In addition, the high percentage related to knowledge demonstrates the consistency of theory-based approaches in changing human behavior through the information that is based on personal beliefs that could motivate or discourage behavior change towards better health [28]. Based on the data gathered from the questions on the risk of RTI infection, 43.1%, 65.3%, and 28.9% of pilgrims were unaware of smokers, diabetic patients, and those in crowded places, respectively, being at risk of contracting the infection. This is very crucial as these pilgrims are at risk and the tendency to get RTIs could be high if the participants have any comorbidity.

As for the complications of RTIs, the majority of the participants knew pneumonia (64.4%) and bronchitis (46.2%) were associated with RTIs. On the other hand, 23.1% and 25.8% of the participants believed that difficulty in breathing and multiorgan failure were complications of RTIs respectively. In a cohort study conducted, pneumonia and bronchitis were reported to be complications associated with RTI in a community setting [29].

Concerning knowledge of the preventive practices, the highest percentage was reported for ensuring a healthy diet (89.8%) in the current study. Similar percentages were observed on receiving vaccination (83.1%), washing hands with hand sanitizers (87.1%), wearing face masks (90.7%) and cough etiquette (68.0%). Almost similar findings were observed in Saudi (61.3%) for the knowledge of vaccination [30]. Majority of French pilgrims during the 2008 Hajj pilgrimage had knowledge of the use of face mask for self-protection (41.3%), the practice of the use of hand disinfectants (2.8%) and social distancing (48.7%) [14]. Knowledge scores were significantly associated with occupation, educational level, previous Umrah experience of the pilgrims, neuromuscular disease, diabetes, and recommended vaccines.

In general, the study participants had overwhelmingly unacceptable attitudes in the present study (93.8%). On the other hand, good knowledge was not reflected by their unacceptable attitudes and practices for some variables in the present study. For instance, 26.2% of the study participants had agreed that vaccination had unpleasant side effects, despite having a good understanding of its usage for prevention. This figure was similar to the figure reported among Umrah pilgrims from Egypt (60.3%) [31]. Preventive practices did not reflect the adequate knowledge reported in this study. In practice, generally, only 16.9% of the pilgrims were shown to have good preventive practices towards respiratory tract infection. However, the majority of the respondents ensured they fitted the face mask properly before using it. Despite having awareness regarding the availability of the recommended vaccines, most of the participants were not immunized against RTIs. However, gender, educational level, previous Hajj experience, and meningococcal vaccine are significantly associated with mean attitude scores. Similarly, gender, occupation, previous Hajj experience were the only factors significantly associated with mean practice scores.

The results obtained from this study showed a weak negative correlation between knowledge and attitude among the Malaysian Hajj and Umrah pilgrims towards the prevention of RTI. However, a previous study reported a positive correlation between knowledge and attitude among pilgrims. It has been mentioned that knowledge helps to improve attitude [31]. Additionally, a negative correlation was observed between knowledge and practice. Attitude and practice among the pilgrims had a significant weak positive correlation. These findings were similar to a previous research report, which found a positive correlation between attitude and practice. The r-value suggested that good attitude leads to positive practice.

This study has some limitations. The study was conducted in one state in Malaysia, and therefore, the results of the research are not representative of the entire population of Malaysia. However, the present study concludes that there is good knowledge among the Hajj and Umrah pilgrims from Malaysia towards prevention of respiratory tract infections, their mode of transmission, and spread. However, the majority of the pilgrims were not fully vaccinated against influenza (24.9%) and pneumococcus (27.6%), as recommended by the health authorities.

## 5. Conclusions

The gap recognized in this study can serve as baseline data to design effective interventions to evaluate the prevention strategies for prevention of RTI. These findings could also provide an insight for the Hajj commission and health authorities to improve the knowledge, attitude, and practice of the pilgrims through health education and a more robust awareness and publicity.

## Figures and Tables

**Table 1:** Characteristics of the study respondents (*n* = 225).

Variables		Mean (SD)	Frequency (%)
Age		46.74 (13.38)	
Gender	Male		74 (32.9)
	Female		151 (67.1)
Ethnicity	Malay		223 (99.1)
	Others		2 (0.9)
Marital status	Single		46 (20.4)
	Married		169 (75.1)
	Divorced/widowed		10 (4.4)
Occupation	Civil servant		95 (42.2)
	Self-employed		27 (12.0)
	Private		14 (6.2)
	Pensioner		41 (18.2)
	Housewife		31 (13.8)
	Student		17 (7.6)
Highest level of education	Bachelor		78 (34.7)
	Diploma		37 (16.4)
	Master		18 (8.0)
	PhD		8 (3.6)
	Secondary school		80 (35.6)
	Primary school		4 (1.8)
History of vaccination	Influenza (flu) vaccine		56 (24.9)
	Pneumococcal vaccine		62 (27.6)
Presence of Co-morbidities	Chronic lung disease		5 (2.2)
	Neuromuscular disease		17 (7.6)
	Allergic rhinitis		12 (5.3)
	Diabetes		31 (13.8)
	Hypertension		55 (24.4)
	Heart disease		2 (0.9)
	Immune deficiency disorders		7 (3.1)
Presence of respiratory illness before departure	Yes		8 (3.6)
	No		217 (96.4)

**Table 2:** Responses to knowledge items.

Questions		Response n (%)	
		Correct Answer	Wrong Answer
K1	Flu-like illnesses are caused by:		
i	Viruses	180 (80)	45 (20.0)
ii	Bacteria	113 (50.2)	112 (49.8)
iii	Allergies	103 (45.8)	122 (54.2)
K2	Flu-like illnesses are spread by:		
i	Water	88 (39.1)	137 (60.7)
ii	Sharing towels with an infected person	116 (51.6)	109 (48.4)
iii	Dust	168 (74.7)	57 (25.3)
iv	Air	192 (85.3)	33 (14.7)
v	Shaking the hands of an infected person with a cough and/or cold	100 (44.4)	125 (55.6)
K3	Flu-like illnesses are spread quickly	152 (67.6)	73 (32.4)
K4	The following persons are at an increased risk of flu-like illnesses:		
i	Senior citizens aged 65 and older	157 (69.8)	68 (30.2)
ii	Smokers	128 (56.9)	97 (43.1)
iii	Asthmatics	163 (72.4)	62 (27.6)
iv	Diabetics	78 (34.7)	147 (65.3)
v	People with arthritis	72 (32.0)	153 (68.0)
vi	Those in crowded places/among a lot of people	160 (71.1)	65 (28.9)
K5	What are the complications of flu-like illnesses?		
i	Pneumonia	145 (64.4)	80 (35.6)
ii	Bronchitis	104 (46.2)	121 (53.8)
iii	Difficulty in breathing	52 (23.1)	173 (76.9)
iv	Multi-organ failure	58 (25.8)	167 (74.2)
K6	The following practices can help protect you from flu-like illnesses:		
i	Ensuring a healthy diet	202 (89.8)	23 (10.2)
ii	Receiving vaccinations	187 (83.1)	38 (16.9)
iii	Washing your hands with hand sanitizers	196 (87.1)	29 (12.9)
iv	Covering your nose with your hands	153 (68.0)	72 (32.0)
v	Wearing a face mask	204 (90.7)	21 (9.3)
K7	The following are reasons for wearing a mask:		
i	Being in crowded places	205 (91.1)	20 (8.9)
ii	Being near people who are coughing	208 (92.4)	17 (7.6)
iii	When I am sick	192 (85.3)	33 (14.7)
K8	A cloth face mask is as effective as a two-ply surgical face mask	51 (22.7)	174 (77.3)
K9	If I am not sick, the used face mask can be stored in a bag for later use	172 (76.4)	53 (23.6)

**Table 3:** Attitude toward RTIs prevention.

Questions		Strongly Agree	Agree	Not Sure	Disagree	Strongly Disagree
1	Since the bird flu, SARS, MERS-COV, and H1N1 crises are over, I no longer need to worry about contracting flu-like illnesses	45 (20.0)	120 (53.3)	26 (11.6)	26 (11.6)	8 (3.6)
2	I am generally opposed to wearing a face mask	66 (29.3)	122 (54.2)	21 (9.3)	13 (5.8)	3 (1.3)
3	Flu vaccinations have unpleasant side effects	22 (9.8)	59 (26.2)	104 (46.2)	25 (11.1)	15 (6.7)
4	I am influenced by negative news about flu vaccines	28 (12.4)	102 (45.3)	63 (28.0)	27 (12.0)	5 (2.2)
5	It is too much trouble to get a flu vaccine	29 (12.9)	105 (46.7)	63 (28.0)	23 (10.2)	5 (2.2)
6	If I have a flu-like illness, I may spread it to others	5 (2.2)	9 (4.0)	52 (23.1)	124 (55.1)	35 (15.6)
7	I feel that someone who has influenza-like illness should:					
i	cover his mouth and nose with his bare hand when coughing or sneezing	1 (0.4)	26 (11.6)	40 (17.8)	87 (38.7)	71 (31.6)
ii	cover his mouth and nose with a handkerchief when coughing or sneezing		8 (3.6)	15 (6.7)	110 (48.9)	92 (40.9)
8	Influenza vaccines protect hajj pilgrims from influenza	1 (0.4)	12 (5.3)	26 (11.6)	127 (56.4)	59 (26.2)
9	Using a hand wash can prevent you from getting flu-like illness		11 (4.9)	47 (20.9)	110 (48.9)	57 (25.3)
10	I think coughs and the flu can be prevented by wearing a mask outside my house	2 (0.9)	16 (7.1)	52 (23.1)	124 (55.1)	31 (13.8)
11	Wearing a well-fitting face mask is effective in preventing flu-like illnesses		14 (6.2)	50 (22.2)	124 (55.1)	37 (16.4)

**Table 4:** Practice related to RTI prevention.

Items		Always	Occasionally	Never
1	I eat vegetables	80 (35.6)	139 (61.8)	6 (2.7)
2	I eat fruits	76 (33.8)	143 (63.6)	6 (2.7)
3	I use soap to wash my hands	99 (44.0)	116 (51.6)	10 (4.4)
4	When wearing a mask, I test it to ensure it fits properly	73 (32.4)	105 (46.7)	47 (20.9)
5	I use disinfectant or disposable wipes or hand gel to wash my hands	39 (17.3)	114 (50.7)	72 (32.0)
6	I use a washable cloth handkerchief to clean my hands	20 (8.9)	106 (47.1)	99 (44.0)
7	I wash my hands after:			
i	touching the personal items of someone who has a cough and/or cold	69 (30.7)	99 (44.0)	57 (25.3)
ii	shaking hands with people who have a cough and/or cold	56 (24.9)	91 (40.4)	78 (34.7)
iii	touching doorknobs	32 (14.2)	74 (32.9)	119 (52.9)
8	I refrain from:			
i	being close to those who cough or sneeze	54 (24.0)	110 (48.9)	61 (27.1)
ii	shaking the hands of those who have a cough and/or cold	37 (16.4)	108 (48.0)	80 (35.6)
iii	often touching my nose	38 (16.9)	103 (45.8)	84 (37.3)
9	I received the flu vaccine	23 (10.2)	103 (45.8)	84 (37.3)

## Supplementary Materials

The following are available online at https://www.mdpi.com/1660-4601/16/22/4569/s1, Figure S1: Correlation knowledge and attitude score, Figure S2: Correlation between knowledge and practice score: Figure S3: Correlation between attitude and practice scores: Table S1: Comparison of Demographic Characteristics and Mean KAP Scores.
