# Supplementary material for: Assessment of Knowledge, Attitude and Practice towards Prevention of Respiratory Tract Infections among Hajj and Umrah Pilgrims from Malaysia in 2018
Source: Int J Environ Res Public Health. 2019 Nov 18;16(22):4569. doi: 10.3390/ijerph16224569 (PMC6888533; doi:10.3390/ijerph16224569)
Supplement: Supplementary file 1 [file ijerph-16-04569-s001.zip › ijerph-573797-SI/Hajj_Questionnaire.docx]

A: KNOWLEDGE OF FLU-LIKE ILLNESSES

*A: PENGETAHUAN PENYAKIT MIRIP INFLUENZA*

Please indicate (*√*) on the space provided accordingly and answer all questions

*Sila nyatakan (√) dalam ruang yang disediakan dan jawab semua soalan.*

| *No.* | | Knowledge  *Pengetahuan* | Yes  *Ya* | No  *Tidak* | I don’t know  *Saya tidak tahu* |
| --- | --- | --- | --- | --- | --- |
| *1* | | Flu-like illnesses are caused by:  *Penyakit mirip influenza (demam selesema) disebabkan oleh:* |  |  |  |
|  | *i* | Virus  *Virus* |  |  |  |
|  | *ii* | Bacteria  *Bakteria* |  |  |  |
|  | *iii* | Allergies  *Alergi* |  |  |  |
| *2* | | Flu-like illnesses are spread by:  *Penyakit mirip influenza (demam selesema) disebarkan oleh:* |  |  |  |
|  | *i* | Water  *Air* |  |  |  |
|  | *ii* | Sharing towels with an infected person  *Berkongsi tuala dengan penghidap* |  |  |  |
|  | *iii* | Dust  *Debu* |  |  |  |
|  | *iv* | Air  *Udara* |  |  |  |
|  | *v* | Shaking the hands of an infected person with a cough and/or cold  *Berjabat tangan dengan pengidap yang batuk atau demam* |  |  |  |
| *3* | | Flu-like illnesses are spread quickly  *Penyakit mirip influenza (demam selesema) tersebar dengan pantas* |  |  |  |
| *4* | | The following persons are at an increased risk of flu-like illnesses:  *Berikut merupakan mereka yang berisiko tinggi mengalami penyakit mirip influenza (demam selsema):* |  |  |  |
|  | *i* | Senior citizens aged 65 and older  *Warga emas berusia 65 tahun ke atas* |  |  |  |
|  | *ii* | Smokers  *Perokok* |  |  |  |
|  | *iii* | Asthmatics  *Pesakit asma* |  |  |  |
|  | *iv* | Diabetics  *Pesakit diabetes* |  |  |  |
|  | *v* | People with arthritis  *Penghidap artritis* |  |  |  |
|  | *vi* | Those in crowded places/among a lot of people  *Mereka yang berada di tempat yang sesak/dalam kalangan ramai orang* |  |  |  |
| *5* | | *Apakah komplikasi penyakit seperti selsema?* |  |  |  |
|  | *i* | Pneumonia  *paru-paru berair* |  |  |  |
|  | *ii* | Difficulty in breathing  *kekejangan* |  |  |  |
|  | *iii* | Bronchitis  *Bronkitis* |  |  |  |
|  | *iv* | Multi-organ failure  *kegagalan pelbagai organ* |  |  |  |
| *6* | | The following practices can help protect you from flu-like illnesses:  *Amalan berikut dapat menghindari anda daripada penyakit mirip influenza (demam selesema):* |  |  |  |
|  | *i* | Ensuring a healthy diet  *Memastikan pemakanan yang sihat* |  |  |  |
|  | *ii* | Receiving vaccinations  *Menerima vaksinasi* |  |  |  |
|  | *iii* | Washing your hands with hand sanitizers  *Membasuh tangan dengan sanitasi* |  |  |  |
|  | *iv* | Covering your nose with your hands  *Menutup mulut dengan tangan* |  |  |  |
|  | *v* | Wearing a face mask  *Memakai topeng muka* |  |  |  |
| *7* | | The following are reasons for wearing a mask:  *Berikut merupakan sebab memakai topeng muka:* |  |  |  |
|  | *i* | Being in crowded places  *Berada di tempat yang sesak* |  |  |  |
|  | *ii* | Being near people who are coughing  *Berada berdekatan orang yang batuk* |  |  |  |
|  | *iii* | When I am sick  *Apabila saya sakit* |  |  |  |
| *8* | | A cloth facial mask is as effective as a 2-ply surgical facial mask  *Topeng wajah kain sama berkesan dengan topeng wajah pembedahan dua lapisan* |  |  |  |
| *9* | | If I am not sick, the used face mask can be stored in a bag for later use  *Jika saya tidak sakit, topeng muka terpakai boleh disimpan dalam beg untuk kegunaan seterusnya* |  |  |  |

**B: ATTITUDE TOWARDS FLU-LIKE ILLNESSES PREVENTION**

The following questions are about the attitude towards prevention of flu-like illnesses is rated on Likert scale ranging from strongly agree to strongly disagree. Please indicate (*√*) on the space provided accordingly and answer all questions

*B: SIKAP TERHADAP PENCEGAHAN PENYAKIT MIRIP INFLUENZA (DEMAM SELESEMA)*

*Soalan berikut adalah mengenai sikap terhadap pencegahan penyakit mirip influenza (demam selesema) yang terkadar di atas skala Likert bermula dengan paling setuju kepada paling tidak. Sila nyatakan (√) pada ruang yang disediakan dan jawab kesemua soalan*

| **No** | | Attitude  *Sikap* | Strongly agree  *Sangat bersetuju* | Agree  *Setuju* | Not sure  *Tidak pasti* | Disgree  *Tidak bersetuju* | Strongly disagree  *Sangat tidak bersetuju* |
| --- | --- | --- | --- | --- | --- | --- | --- |
| 1 | | Since the bird flu, SARS, MERS-COV and H1N1 crises are over, I no longer need to worry about contracting flu-like illnesses  *Sejak krisis selesema burung, SARS, MERS-COV and H1N1 berakhir, saya sudah tidak perlu bimbang mendapat penyakit mirip influenza (demam selesema/influenza like illness)* |  |  |  |  |  |
| 2 | | I am generally opposed to wearing a face mask  *Secara amnya saya menentang penggunaan penutup hidung dan mulut* |  |  |  |  |  |
| 3 | | Flu vaccinations have unpleasant side effects  *Vaksinasi influenza mengakibatkan kesan sampingan yang tidak selesa* |  |  |  |  |  |
| 4 | | I am influence by negative news about flu vaccines  *Saya dipengaruhi oleh berita negatif mengenai vaksin selsema* |  |  |  |  |  |
| 5 | | It is too much trouble to get a flu vaccine  *Agak susah untuk mendapatkan vaksin influenza* |  |  |  |  |  |
| 6 | | If I have a flu-like illness, I may spread it to others  *Jika saya menghidap penyakit mirip influenza (demam selesema/influenza like illness), saya mungkin menjangkiti orang lain* |  |  |  |  |  |
| 7 | | I feel that someone that have influenza-like illness should:  *Seseorang yang mengalami penyakit mirip influenza (demam selesema/influenza like illness) haruslah*: |  |  |  |  |  |
|  | i | cover his mouth and nose with his bare hand when coughing or sneezing  *Menutup mulut dan hidung dengan tangannya apabila batuk atau bersin* |  |  |  |  |  |
|  | ii | cover his mouth and nose with a handkerchief when coughing or sneezing  *Menutup mulut dan hidung dengan sapu apabila batuk atau bersin* |  |  |  |  |  |
| 8 | | Influenza vaccines protects hajj pilgrims from influenza  *Vaksin influenza melindungi jemaah haji daripada demam selesema* |  |  |  |  |  |
| 9 | | Using a hand wash can prevent you from getting flu like illness  *Penggunaan sanitasi tangan dapat mencegah anda daripada menghidap penyakit mirip influenza (demam selesema/influenza like illness)* |  |  |  |  |  |
| 10 | | I think coughs and the flu can be prevented by wearing a mask outside my house  *Saya rasa penyakit mirip influenza (demam selesema/influenza like illness) boleh dicegah dengan pemakaian sungkup muka di luar rumah* |  |  |  |  |  |
| 11 | | Wearing a well-fitting face mask is effective in preventing flu-like illnesses  *Memakai sungkup muka yang betul-betul sesuai pada wajah adalah berkesan untuk mencegah penyakit seperti selsema* |  |  |  |  |  |

**C: PREVENTION PRACTICES TOWARDS FLU-LIKE ILLNESSES**

The following questions are about practice towards flu-like illnesses prevention. Please indicate (*√*) on the space provided accordingly and answer all questions

*C: AMALAN PENCEGAHAN TERHADAP PENYAKIT MIRIP INFLUENZA (DEMAM SELESEMA)*

*Soalan berikut adalah berkenaan dengan amalan terhadap pencegahan penyakit mirip influenza (demam selesema). Sila nyatakan (√) pada ruang yang disediakan dan jawab kesemua soalan*

| *No* | | Practice  *Amalan* | Always  *Sentiasa* | Occasional  *Selalunya* | *Never*  *Tidak pernah* |
| --- | --- | --- | --- | --- | --- |
| 1 | | I eat vegetables  *Saya makan sayur* |  |  |  |
| 2 | | I eat fruits  *Saya makan buah-buahan* |  |  |  |
| 3 | | I use soap to wash my hands  *Saya menggunakan sabun untuk membasuh tangan saya* |  |  |  |
| 4 | | When wearing a mask, I test it to ensure it fits properly  *Ketika memakai penutup hidung dan mulut (face mask), saya memastikan ianya benar-benar kemas* |  |  |  |
| 5 | | I use disinfectant or disposable wipes or hand gel to wash my hands  *Saya menggunakan penyahinfektan atau pengelap pakai buang atau gel tangan untuk membasuh tangan saya* |  |  |  |
| 6 | | I use a washable cloth handkerchief to clean my hands  *Saya menggunakan sapu tangan yang boleh dibasuh untuk membasuh tangan saya* |  |  |  |
| 7 | | I wash my hands after:  *Saya membasuh tangan saya:* |  |  |  |
|  | i | touching the personal items of someone who has a cough and/or cold  *Selepas menyentuh barang peribadi seseorang yang mengalami batuk atau selsema* |  |  |  |
|  | ii | shaking hands with people who have a cough and/or cold  *Selepas berjabat tangan dengan seseorang yang mengalami batuk atau selsema* |  |  |  |
|  | iii | touching door knobs  *Selepas menyentuh tombol pintu* |  |  |  |
| 8 | | I refrain from:  Saya menghindari daripada: |  |  |  |
|  | i | being close to those who cough or sneeze  *berada rapat dengan orang yang batuk atau selesema* |  |  |  |
|  | ii | shaking the hands of those who have a cough and/or cold  *berjabat tangan dengan orang yang batuk atau selesema* |  |  |  |
|  | iii | often touching my nose  *kerap menyentuh hidung saya* |  |  |  |
| 9 | | I received the flu vaccine  *Saya menerima vaksin selsema* |  |  |  |

**TERIMA KASIH**
