# Supplementary material for: Assessment of Knowledge, Attitude and Practice towards Prevention of Respiratory Tract Infections among Hajj and Umrah Pilgrims from Malaysia in 2018
Source: Int J Environ Res Public Health. 2019 Nov 18;16(22):4569. doi: 10.3390/ijerph16224569 (PMC6888533; doi:10.3390/ijerph16224569)
Supplement: Supplementary file 1 [file ijerph-16-04569-s001.zip › ijerph-573797-SI/Supplementary Table IJEHPH.docx]

Table 1. Comparison of Demographic Characteristics and Mean KAP Scores.

| **Variables** | **n (225)** | **Knowledge Score** | | **Attitude Score** | | **Practice Score** | |
| --- | --- | --- | --- | --- | --- | --- | --- |
|  |  | Mean (SD) | *p*-value | Mean (SD) | *p*-value | Mean (SD) | *p*-value |
| Gender |  |  |  |  |  |  |  |
| Female | 151 | 14.75 (6.66) | <0.001 | 32.17 (4.719) | 0.030 | 25.64 (4.978) | 0.156 |
| Male | 74 | 19.12 (4.78) |  | 33.62 (4.610) |  | 24.64 (4.903) |  |
| Ethnicity |  |  |  |  |  |  |  |
| Malay | 223 | 17.65 (5.82) | 0.295 | 32.61 (4.710) | 0.248 | 25.31 (4.981) | 0.818 |
| Indian |  |  |  |  |  |  |  |
| Others | 2 | 22.00 (7.07) |  | 36.50 (6.363) |  | 24.50 (4.965) |  |
| Marital status |  |  |  |  |  |  |  |
| Single | 46 | 17.97 (4.53) |  | 32.82 (4.403) | 0.905 | 24.89 (5.429) | 0.478 |
| Married | 169 | 17.04 (6.18) |  | 32.63 (4.863) |  | 25.32 (4.835) |  |
| Divorced/widow | 10 | 21.20 (3.99) |  | 32.10 (4.040) |  | 27.00 (5.077) |  |
| Occupation |  |  |  |  |  |  |  |
| Civil servant | 95 | 18.46 (5.613) | 0.031 | 32.92 (4.798) |  | 25.53 (4.571) | 0.920 |
| Self employed | 27 | 17.25 (5.578) |  | 33.88 (3.017) |  | 24.74 (5.755) |  |
| Private sector | 14 | 16.00 (6.025) |  | 32.64 (4.716) |  | 25.71 (5.837) |  |
| Pensioner | 41 | 15.29 (7.274) |  | 32.39 (5.607) |  | 24.68 (5.012) |  |
| House wife | 31 | 19.03 (4.936) |  | 31.32 (5.325) |  | 25.55 (4.871) |  |
| Student | 17 | 18.76 (2.795) |  | 32.17 (2.351) |  | 25.71 (5.599) |  |
| Education level |  |  |  |  |  |  |  |
| Bachelor | 78 | 17.92 (4.858) | <0.001 | 32.83 (4.467) | 0.667 | 25.03 (4.570) | 0.390 |
| Diploma | 37 | 19.32 (6.083) |  | 33.59 (3.499) |  | 26.51 (5.516) |  |
| Master | 18 | 20.44 (3.221) |  | 32.72 (4.896) |  | 26.39 (4.996) |  |
| PhD | 8 | 21.87 (3.720) |  | 32.62 (3.248) |  | 26.50 (4.899) |  |
| Secondary | 80 | 15.41 (6.444) |  | 31.98 (5.542) |  | 24.64 (5.107) |  |
| Primary | 4 | 22.75 (2.986) |  | 33.25 (3.593) |  | 25.75 (3.500) |  |
| Previous Hajj experience |  |  |  |  |  |  |  |
| Yes | 42 | 17.64 (5.873) | 0.814 | 30.90 (6.135) | 0.008 | 26.43 (5.592) | 0.105 |
| No | 183 | 17.64 (5.842) |  | 33.04 (4.255) |  | 25.05 (4.789) |  |
| Previous Umrah experience |  |  |  |  |  |  |  |
| Yes | 61 | 18.34 (5.778) | 0.305 | 32.32 (5.455) | 0.535 | 26.02 (4.842) | 0.192 |
| No | 164 | 17.44 (5.855) |  | 32.76 (4.433) |  | 25.04 (4.999) |  |
